# Pityriasis lichenoides presented with skin rash mimicking Urticaria: A case report

**DOI:** 10.5339/qmj.2023.sqac.7

**Published:** 2023-11-19

**Authors:** Dalal Madawi, Hassan Mobayed

**Affiliations:** ^1^Allergy and Immunology Division, Department of Medicine, Hamad Medical Corporation, Doha, Qatar Email: DMudawi1@hamad.qa

**Keywords:** Pityriasis lichenoides, urticaria

## Abstract

Background: Acute urticaria is urticaria with or without angioedema that is present for less than six weeks, while chronic urticaria is present for more than six weeks.

Pityriasis lichenoides (PL) is a benign cutaneous inflammatory disease of unknown etiology. Acute PL typically resolves within a few weeks, while chronic PL lasts several months. The skin rash of PL may resemble the rash of other conditions, so the distinction is essential and depends on history and physical examination and is confirmed by skin biopsy.

Case report: A 64-year-old gentleman presented with seven days history of generalized itchy skin (hives). Individual lesions last 24-48 hours and do not leave pigmentation or scarring. No systemic involvement. No specific triggers, with two previous similar episodes 30 years and 20 years ago. Levocetirizine, 5 mg tablet, was prescribed, and he was instructed to increase the dose to 4 tablets daily if needed. On reassessing the patient after ten days, he did not respond well. The rash was different from the initial one, with individual lesions lasting for five days or more, so he was referred to a dermatologist for a skin biopsy. Basic investigations were normal. Performing skin biopsy is needed to exclude other pathologies. Skin biopsy showed pathological changes of lichenoid dermatitis compatible with pityriasis lichenoides et varioliformis acuta (PLEVA). He has been treated with azithromycin 250 mg daily for three weeks with rapid and complete resolution without scaring.

Conclusion: Urticarial rash may mimic the skin rash of other conditions. Detailed serial history and physical examination are warranted to exclude other diagnoses. Skin biopsy is needed when diagnosing conditions other than urticaria are suspected.

## Figures and Tables

**Figure 1.**
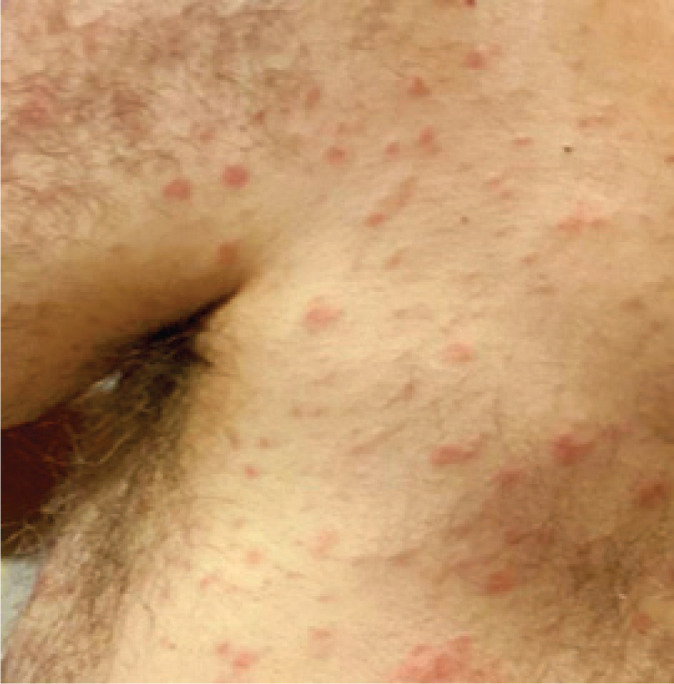
Well-defined erythematous papules in the back.
